# The Incorporation of Amplified Metal-Enhanced Fluorescence in a CMOS-Based Biosensor Increased the Detection Sensitivity of a DNA Marker of the Pathogenic Fungus *Colletotrichum gloeosporioides*

**DOI:** 10.3390/bios10120204

**Published:** 2020-12-13

**Authors:** Dorin Harpaz, Noam Alkan, Evgeni Eltzov

**Affiliations:** 1 Institute of Plant Sciences and Genetics in Agriculture, Faculty of Agriculture, Food and Environment, The Hebrew University of Jerusalem, Rehovot 76100, Israel; dorin.harpaz@mail.huji.ac.il; 2 Institute of Postharvest and Food Science, Department of Postharvest Science, Volcani Center, Agricultural Research Organization, Rishon LeZion 7505101, Israel; noamal@agri.gov.il

**Keywords:** pathogenic fungus, *Colletotrichum gloeosporioides*, metal-enhanced fluorescence, silver deposition, CMOS biosensor, postharvest system

## Abstract

Half of the global agricultural fresh produce is lost, mainly because of rots that are caused by various pathogenic fungi. In this study, a complementary metal-oxide-semiconductor (CMOS)-based biosensor was developed, which integrates specific DNA strands that allow the detection of enoyl-CoA-hydratase/isomerase, which is a quiescent marker of *Colletotrichum gloeosporioides* fungi. The developed biosensor mechanism is based on the metal-enhanced fluorescence (MEF) phenomenon, which is amplified by depositing silver onto a glass surface. A surface DNA strand is then immobilized on the surface, and in the presence of the target mRNA within the sample, the reporter DNA strand that is linked to horseradish peroxidase (HRP) enzyme will also bind to it. The light signal that is later produced from the HRP enzyme and its substrate is enhanced and detected by the coupled CMOS sensor. Several parameters that affect the silver-deposition procedure were examined, including silver solution temperature and volume, heating mode, and the tank material. Moreover, the effect of blocking treatment (skim milk or bovine serum albumin (BSA)) on the silver-layer stability and nonspecific DNA absorption was tested. Most importantly, the effect of the deposition reaction duration on the silver-layer formation and the MEF amplification was also investigated. In the study findings a preferred silver-deposition reaction duration was identified as 5–8 min, which increased the deposition of silver on the glass surface up to 13-times, and also resulted in the amplification of the MEF phenomenon with a maximum light signal of 50 relative light units (RLU). It was found that MEF can be amplified by a customized silver-deposition procedure that results in increased detection sensitivity. The implementation of the improved conditions increased the biosensor sensitivity to 3.3 nM (4500 RLU) with a higher detected light signal as compared to the initial protocol (400 RLU). Moreover, the light signal was amplified 18.75-, 11.11-, 5.5-, 11.25-, and 3.75-times in the improved protocol for all the tested concentrations of the target DNA strand of 1000, 100, 10, 3.3, and 2 nM, respectively. The developed biosensor system may allow the detection of the pathogenic fungus in postharvest produce and determine its pathogenicity state.

## 1. Introduction

Worldwide, one billion people suffer from malnutrition due to lack of food supplies, and two billion people suffer from a lack of essential nutrients and vitamins in their diet [1]. Over the past few years, remarkable progress has been made in increasing the production of food as an approach for food security. However, there is still an unmet need to reduce food losses [2]. According to studies that were conducted by various international and national organizations, mostly led by the Food and Agriculture Organization of the United Nations (FAO), an estimation of one-third of all the food and almost half of all the fruit and vegetables that are produced on the planet, are lost and not consumed [3]. Moreover, half of the harvested food (1.3 billion tons a year) is lost mostly because of rots that are caused by pathogenic fungi [4]. Reducing the rate of food loss is one of the possible solutions to achieving food security [5]. In addition, the growth in the world population implies additional challenges. The U.N. predicts that the world global population will reach 9.7 billion people by 2050, compared to 7.3 billion people in 2015. Countries around the world are currently focused on efforts to increase their food supply in order to meet the growing demand [6]. Moreover, pathogenic fungi produce toxic compounds that are later consumed and may cause health problems in animals and humans [7]. Therefore, for both food safety and security, it is important to monitor the presence of pathogenic fungi in harvested produce.

Various genera of pathogenic fungi may cause decay in harvested fresh produce [8,9,10]. The fungi usually remain quiescent in immature fruit and only switch to their pathogenic state after ripening. Such quiescent infections are microscopic, and without external signs that can be visually detected during the supply chain [11,12,13]. Thus, there is a need to develop sensing platforms that will be able to detect fungal infections in both their quiescent and pathogenic states. *Colletotrichum gloeosporioides* (*C. gloeosporioides)* is one of the most common pathogenic fungi in harvested fruit such as mango, avocado, and strawberry, with 470 well-known host genera [14]. *C. gloeosporioides* has three identified states, including penetration (appressoria), latent stage (quiescence), and pathogenic (necrotrophic) state [13]. After fruit ripening, *C. gloeosporioides* switches from the quiescent state to the pathogenic state that causes anthracnose disease in the fruit [14]. Interestingly, during the quiescent state, most of the transcripts are down-regulated, while specific transcripts are upregulated in *C. gloeosporioides* in the pathogenic state, which can be used as markers. Conventional technologies already exist for the detection of the pathogenic fungus in harvested produce, such as polymerase chain reaction (PCR) and enzyme-linked immunosorbent assay (ELISA) [15]. These technologies provide reliable and sensitive results; however, they are costly and require complicated measurement processes that makes them less suitable for use in agricultural applications. Therefore, practical solutions are still lacking and are required in order to allow real-time monitoring of pathogenic fungi and potential decay development in fruits and vegetables during the supply chain, retail, and consumer storage.

Several biosensing approaches are currently being investigated in order to monitor pathogens in plants [16]. A preferred approach for detecting pathogenic fungus may be a biosensor for RNA detection. A biosensor is a self-contained bionic integrated device that includes a biological recognition element (enzyme, antibody, receptors, and microorganisms), which can respond in a concentration-dependent manner to monitor a biochemical species. Biosensor platforms offer advantages when compared to conventional analytical methods—they are miniaturized and portable, which permits their use as on-site devices, they are also often cheap, simple to use, and do not require any sample preparation. Various sensing technologies were tested for DNA detection, including optical [17], mass balance [18,19], and quartz crystal microbalance [20,21]. Technological advances have led to the development of miniaturized and sensitive photodetectors termed complementary metal-oxide-semiconductor (CMOS) sensors [22]. CMOS circuits can be found in multiple electronic components, including microprocessors, batteries, and digital camera image sensors. A CMOS image sensor usually contains an electronic rolling shutter that eliminates the need for a mechanical shutter except in certain cases, and each bucket can be read independently to the output. These advantages allow CMOS image sensors to be used in some of the highest-specification industrial control devices and the finest cameras [23]. CMOS-based biosensors were developed for the detection of bacteria [24,25], water, and air toxicants [26,27], coupled with PCR [28,29], and used in food safety [30,31,32,33,34]. Only a few CMOS-based biosensor platforms were developed for use in agriculture applications [35,36].

In this study, a complementary CMOS-based biosensor was developed, which integrates specific DNA strands that allow the detection of enoyl-CoA-hydratase/isomerase, which is a quiescent marker of *C. gloeosporioides* fungi. The developed biosensor mechanism is based on the metal-enhanced fluorescence (MEF) phenomenon, which is amplified by depositing silver onto a glass surface [37]. A surface DNA strand is then immobilized on the surface, and in the presence of the target mRNA within the sample, the reporter DNA strand that is linked to horseradish peroxidase (HRP) enzyme will also bind to it. The light signal that is later produced from the HRP enzyme and its substrate is enhanced and detected by the coupled CMOS sensor. Several parameters that affect the silver-deposition procedure were examined, including silver solution temperature and volume, heating mode, and the tank material. Moreover, the effect of blocking treatment (skim milk or bovine serum albumin (BSA)) on the silver-layer stability and nonspecific DNA absorption was tested. Importantly, the effect of the deposition reaction duration on the silver-layer formation and MEF amplification was also investigated. The developed biosensor system may allow the detection of the pathogenic fungi even at their quiescent state in postharvest produce and determine its pathogenicity state.

## 2. Materials and Methods

### 2.1. Materials

Hydrochloric acid (HCl) 37% (#953503), hydrogen peroxide (H_2_O_2_) (#2186-1), and sulfuric acid (H_2_SO_4_) (#9681-03) were purchased from John Townsend (J.T.) Baker (Phillipsburg, NY, USA). Methyl alcohol (#6712) was purchased from Macron (Center Valley, PA, USA). 3-glycidoxypropyltrimethoxysilane (GPTMS), silane (#440,167), Tris-EDTA (#101,903,432), skim milk powder (#70,166), and bovine serum albumin (BSA) (#A7906) were purchased from Sigma-Aldrich (Rehovot, Israel). Clarity™ western ECL substrate and peroxide + luminol (#1705061) was purchased from Bio-Rad (Rishon Le Zion, Israel). Silver nitrate coating: D-Glucose (#FG/0500/60), silver nitrate (#7761-88-8), and sodium hydroxide (#FS/4880/60) were purchased from Fisher Chemical (Fair Lawn, NJ, USA). Ammonium hydroxide (#033285.1) was purchased from Alfa Aesar (Lancashire, UK). DNA strands: The surface strand (5′-ATG CAC CGT AGC GAC CAG AG-3′-SH) and the target strand of the quiescent marker (Cgl_00014454, enoyl-coA-hydratase/isomerase—5′-CCC AAG CTC ATA GGA CTG TCT AAG GCG AGC CAC GTC ACG ACC ACT GGA GAC GTG TAT CCC GTC ACC GAT CCA CTC GTC AAT GGG CTG TTC TCA AAG TTG CTT CCC ACG CCT CAA CAC ACA GTC-3′) were purchased from HyLabs (Rehovot, Israel). The reporter strand (horseradish peroxidase (HRP)—5′-TGA GGC GTG GGA AGC AAC-3′) of reverse complementary of the quiescent marker (Cgl_00014454, enoyl-coA-hydratase/isomerase) was purchased from Eurogentec (Seraing, Belgium).

### 2.2. Surface Activation and Silver-Deposition Procedure

The biosensor surface was formed on 350 µL flat bottom glass tubes (CSI, # I025-630). The glass tubes were firstly cleaned by incubation in MeOH: HCl solution (ratio 1:1 (*v*/*v*)) at room temperature for 20 min and then placed in a sonication bath (JK-OCD30A, MRC, Holon, Israel) dipped in deionized water (DI) for 20 min. The cleaning step was conducted in order to clean any inorganic and organic surface contaminants. After cleaning, the glass tubes were further activated by incubation in piranha solution (H_2_O_2_: H_2_SO_4_ (3:7)) at 90 °C for 60 min, in order to improve the surface hydrophilicity by hydroxylation. The tubes were then rinsed with DI and dried with N_2_. In the next silanization step, the glass tubes were incubated in 3-glycidoxypropyltrimethoxysilane at 60 °C for 60 min. After that, the silanized glass tubes were rinsed with DI water and dried. Then, the silver liquid deposition proceeded as previously described [37], based on the Tollens’ reagent, which is a colorless solution of a diamminesilver(I) complex that is created by a mixture of silver nitrate, ammonia, and alkaline. Next, 500 µL fresh 5% (*w*/*v*) NaOH solution was added into a fast-stirring silver nitrate solution (0.22 g in 26 mL of DI). The NaOH–silver nitrate solution was continuously mixed until the formation of dark-brownish precipitates was visible, then, less than 1 mL of ammonium hydroxide was added to the deposition solution until the precipitates were dissolved. The clear solution was cooled to 5 °C by placing it in an ice bath. After, the glass tubes were placed into the silver deposition solution, and a fresh D-glucose solution (0.35 g in 4 mL of DI) was added, while the mixture was stirred for 2 min. Then, the solution was warmed to 30 °C. The glass tubes were incubated inside the silver deposition solution until a silver layer was formed on the glass surface. The tubes were then washed with DI and dried. The efficiency of the silver deposition on the glass tube surfaces was evaluated by spectrophotometry. The silver-deposited glass tubes were then placed in 1.2 mL plastic cuvettes (Srastedt, Germany) that were modified by creating a hole to hold the tested tubes and by cutting the sides of the cuvette for better light propagation. The optical density of the silver-deposited glass tubes was determined by an Ultrospec 2100 pro spectrometer (Amersham Biosciences) at 420 nm.

### 2.3. DNA Immobilization

The silanized glass tubes, after silver deposition, were then used for the immobilization of the surface DNA strand. The tubes were incubated in a 20 µL 1 µM surface-DNA-strand solution diluted with Tris-EDTA buffer for 1 h at room temperature in the dark. After the immobilization step of the surface DNA strand, the biofunctionalized glass tubes were washed with Tris-EDTA buffer and incubated in a blocking agent solution (skim milk or BSA). The blocked glass tubes were then exposed to 20 µL 1 µM of target- and reporter DNA strands. Conventional PCR proceeded with a Veriti 96-well thermal cycler (Applied Biosystems) by applying one thermal program cycle (60 s at 90 °C, then 30 s at 75 °C, then 30 s at 65 °C, and finally 30 s at 53 °C). The DNA quality and quantity were then measured by spectrophotometry (NanoDrop 1000 Spectrophotometer, Thermo Scientific, Wilmington, DE, USA). After, the exposed glass tubes were washed with Tris-EDTA buffer and placed on the CMOS sensor or in IVIS (IVIS-100, Perkin Elmer, Waltham, MA, USA) for the monitoring of the light signal.

### 2.4. CMOS-Based Biosensor-System Setup

**Figure 1:**
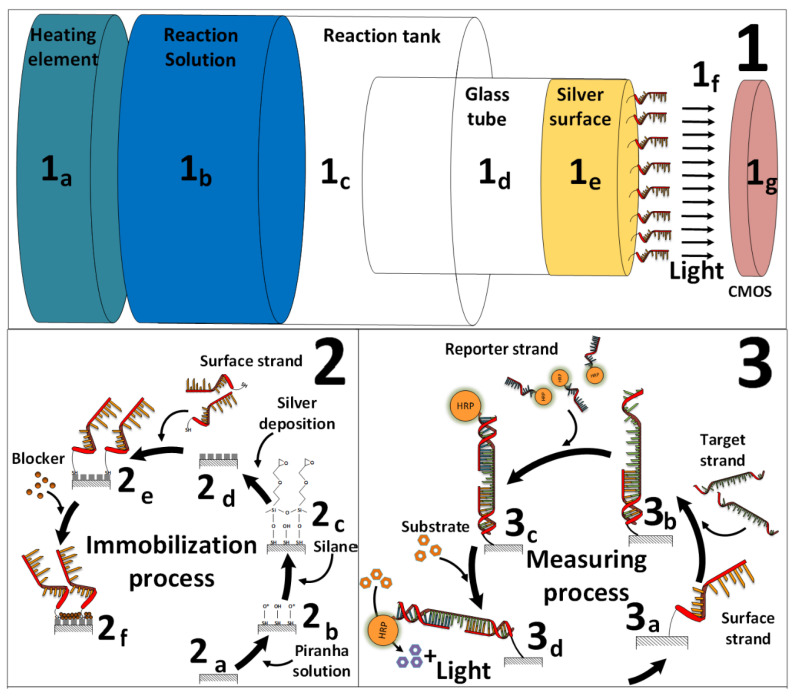
Schematic presentation of the CMOS-based biosensor. (**1**) Different sections of the complementary metal-oxide-semiconductor (CMOS)-based biosensor platform: (**1a**) heating element for the silver-deposition reaction; (**1b**) silver reaction solution that is used to polymerize the silver layer above the glass surface; (**1c**) reaction tank where the polymerization reaction occurs; (**1d**) glass tube where the deposition and measurement processes occur; (**1e**) silver surface nanolayer with immobilized surface DNA strands; (**1f**) horseradish peroxidase (HRP) enzymatic reaction produces a measurable light signal, amplified with metal-enhanced phenomenon (MEF); (**1g**) CMOS photodetector that is coupled near the light signal from positive samples and transforms it into measurable electrical current. (**2**) The DNA immobilization process onto the glass surface: (**2a**) unmodified glass surface; (**2b**) activation with piranha solution; (**2c**) silanization; (**2d**) nucleation points for further silver deposition; (**2e**) immobilization of surface DNA strands; (**2f**) blocking agent (skim milk or bovine serum albumin (BSA)). (**3**) The measurement process where the surface DNA strand is immobilized onto the silver-deposited glass: (**3a**) exposure to the target strand; (**3b**) exposure to the reporter strand that is linked to HRP; (**3c**) the strands specifically anneal into one complex; (**3d**) addition of substrate ((1:1) luminol: H_2_O_2_ (*v*/*v*)) produces a measurable light signal.

**Figure 2:**
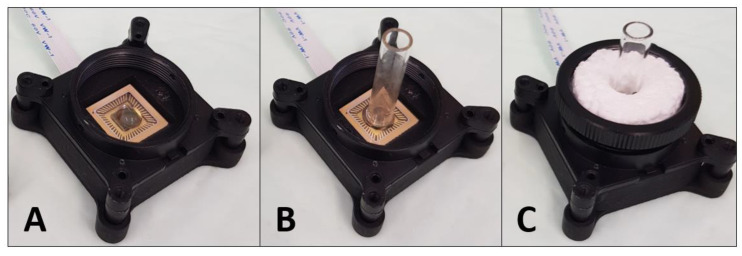
Picture of the CMOS-based biosensor system setup. (**A**) the enzymatic reaction of the horseradish peroxidase (HRP) enzyme was activated by the deposition of a 20 µL substrate solution ((1:1) luminol: H_2_O_2_ (*v*/*v*)), (**B**) while the glass tubes were still placed above the CMOS sensor. (**C**) A home-made holder from Styrofoam was integrated with the CMOS-based biosensor system setup, which prevents the movement of the glass tube during the measurement process. The light signal was directly monitored and detected by the CMOS sensor.

After exposure to the DNA strands, the treated glass tubes were placed onto the CMOS sensor to monitor the light signal. The coupling with a CMOS sensor enables a setup design of the biosensor system that is suitable to be operated directly in the field (Figure 1 and Figure 2). The CMOS sensor (ULS 24 solution kit by Anitoa (Palo Alto, CA, USA)) was then placed in a light-tight box to prevent any possible interference from the surrounding light. The 0.18 µm CMOS imager has an ultra-low-light sensitivity, with a detection threshold of ~3.0 × 10^−6^ lux, low dark current (high SnR > 13dB at detection threshold), 12-bit analog-to-digital converter (ADC), integration time of 100 µs–100 s (controlled by the software), wide dynamic range (~85 dB), and a digital interface through serial peripheral interface (SPI). The CMOS imager size is 4.9 mm × 4.8 mm, with a sensing area of 3.6 mm × 3.6 mm [38]. Specific software was developed by Anitoa company to collect and analyze the light signal data in real-time. The enzymatic reaction of the HRP enzyme was activated by the deposition of a 20 µL substrate solution ((1:1) luminol: H_2_O_2_ (*v*/*v*)), while the glass tubes were still placed above the CMOS sensor. In addition, a home-made holder from Styrofoam was integrated with the CMOS-based biosensor system setup, which prevented the movement of the glass tube during the measurement process. The light signal was directly monitored and detected by the CMOS sensor.

### 2.5. Parameters that Influence the Silver-Deposition Procedure Efficiency

Various parameters that influence the silver-deposition procedure were examined. First, the effect of the reaction temperature was examined by incubating the glass tubes in the 26 mL silver solution for 9 min at two different temperatures of 30 °C or 35 °C. Second, the effect of the heating mode was tested, by heating the glass tubes to 35 °C either on a heating plate (MHK-4D, Fried Electric, Israel) or in a water bath (Pura 10, Julabo, Germany). Third, the effect of the silver solution volume was tested; three solution volumes of 4, 15, and 26 mL were used. Fourth, the effect of a blocking agent was examined, the modified glass tubes were incubated for 1 h in 4 mL of two different blocking-agent solutions (1, 2, 3, 4, and 5% (*w*/*v*)) skim milk or (1, 2, 3, and 4% (*w*/*v*)) BSA diluted in PBS (0.05% (*v*/*v*) Tween 20) (PBST)). As a control, the modified tubes were incubated with PBST only. Then, the glass tubes were rinsed ten times with 1 mL PBST, and after each washing step, the stability of the silver layer was tested. Then, the effect of the blocking procedure on the nonspecific DNA absorption on the glass surface was tested. The glass tubes without immobilized DNA were blocked either with BSA (1, 2, or 3% (*w*/*v*)) or skim milk (1, 2, 3, or 4% (*w*/*v*)), and then incubated for 1 h with the reporter DNA strand. Alternatively, the glass tubes with the immobilized surface DNA strand were blocked with the same BSA or skim milk concentrations, and then exposed to the target and reporter DNA strands. Lastly, the effect of the deposition time on the MEF phenomenon was examined. The silver layer on the glass tubes was polymerized using optimum immobilization conditions (heating 4 mL of the silver deposition solution in a water bath at 35 °C) for different silver-deposition reaction durations (1, 2, 3, 4, 5, 6, 7, 8, 9, 10, 11, and 12 min). Then, the glass tubes were rinsed, treated with a blocking agent of 1% (*w*/*v*) skim milk, and exposed to the target and reporter DNA strands. In addition, scanning electron microscopy (SEM) characterization was conducted on the effect of the deposition time on the formation of silver nano-islands. The surface morphology was characterized by an SEM model MIRA3 from TESCAN (Brno-Kohoutovice, Czech Republic), at a 5 kV accelerating voltage. Before imaging, a thin layer of palladium gold was deposited onto the samples in order to render them electrically conductive and to avoid potential surface charging by the electron beam.

### 2.6. Sensitivity and Reproducibility of the System

The detection sensitivity was tested for different concentrations of the quiescent marker of *Colletotrichum gloeosporioides*, a specific sequence of enoyl-CoA-hydratase/isomerase (Cgl_00014454). The sensitivity of the CMOS-based biosensor system was examined by comparing both the initial and the improved silver-deposition procedures. The initial procedure included a 24-mL silver reaction solution heated at 35 °C on the heater plate for 9 min. While in the improved protocol the 4-mL silver reaction solution was heated at 35 °C in a water bath for 5 min. Then, in both cases, the glass tubes were immobilized with 20 µL 1 µM of surface DNA strands diluted with Tris-EDTA buffer and incubated for 1 h at room temperature in the dark. After washing, the tubes were then treated with a blocking agent solution of 1% (*w*/*v*) skim milk. Next, the glass tubes were incubated with 20 µL of different target DNA strand solution concentrations (0.2, 0.25, 0.3, 0.5, 1, 10, and 100 nM). The glass tubes were then exposed to 20 µL 1 µM of reporter DNA strands, and conventional PCR proceeded. Then, the glass tubes were rinsed, dried, and placed onto the CMOS sensor for light-signal measurement, after the addition of 20 µL substrate solution ((1:1) luminol: H_2_O_2_ (*v*/*v*)). In order to test the system reproducibility, 30 separate biosensor setups were examined for each tested parameter. Two-way repeated-measures analysis of variance (ANOVA) was employed for the evaluation of the dispersion between different tested parameters.

## 3. Results and Discussion

### 3.1. Effect of Different Deposition Conditions on the Efficiency of the Silver-Layer Formation

**Figure 3:**
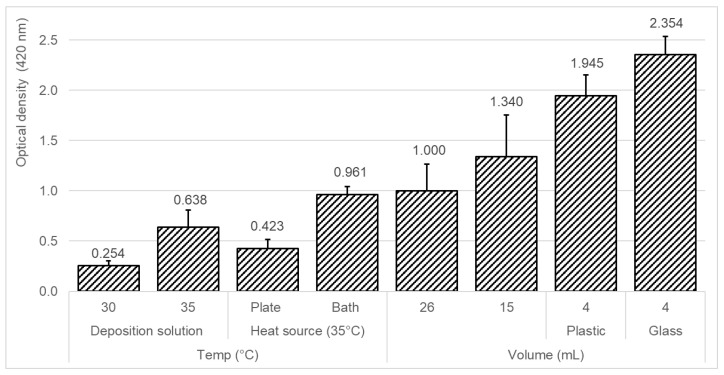
Effect of different deposition conditions on the efficiency of the silver-layer formation. Various parameters were examined including silver solution temperature and volume, heating mode, and the tank material. The silver-deposition reaction duration was 9 min in all the tested parameters.

The formation of a silver layer on a glass surface has been well-known for several decades and described in multiple studies [39,40,41,42,43,44] and patents [45,46,47] by applying various techniques. The most-common silver-deposition reaction is based on Tollens’ reagent, which is a colorless solution of a diamminesilver(I) complex that is created by a mixture of silver nitrate, ammonia, and alkaline [48,49,50,51,52,53]. The silver-layer formation is highly influenced by the various conditions of the deposition procedure. The effects of different parameters on the efficiency of the silver-layer formation were examined, including silver solution temperature and volume, heating mode, and tank material (Figure 3). The optical density (OD) at 420 nm was compared as an indication of the silver-layer formation. Higher optical density values are equivalent to increased silver that is deposited on the glass surface. From the results, generally, the silver-deposition procedure was more efficient in the cases of lower deposition solution volumes and by heating the deposition solution with a water bath as the heating source to a higher temperature. Two silver solution temperatures were tested, and a temperature of 35 °C (OD_420 nm_ = 0.638) showed an increased optical density as compared to a temperature of 30 °C (OD_420 nm_ = 0.254), indicating a 2.5-times increased deposition of silver. Among the two tested heat sources, a 2.3-times increased amount of silver was deposited in the case of a water bath (OD_420 nm_ = 0.961) as a heating source as compared to the plate heater (OD_420 nm_ = 0.423). Moreover, three different silver deposition solution volumes were examined of 4, 15, and 26 mL. Among the tested solution volumes, 4 mL (plastic: OD_420 nm_ = 1.945 and glass: OD_420 nm_ = 2.354) showed a 1.76-times and 2.35-times increased deposition of silver, as compared to 15 mL (OD_420 nm_ = 1.340) and 26 mL (OD_420 nm_ = 1.000), respectively. A possible explanation may be centered around the solution temperature because the silver-deposition procedure is highly dependent on the solution temperature. Higher silver deposition solution volumes require a longer heating time; therefore, in a constant deposition time of 9 min, higher solution volumes may result in less deposition of silver. In addition, in the case of a 4-mL silver deposition solution, more silver was deposited when the tank material was glass (OD_420 nm_ = 2.354), as compared to plastic (OD_420 nm_ = 1.945). A possible reason may be the difference in the thermal conductivity coefficients (TCC (between the glass (1.82 (Btu/(h ft °F)) and the polypropylene plastic (0.69 (Btu/(h ft °F))). The higher TCC of glass may result in faster heating of the silver deposition solution, and therefore, may also result in a more-efficient silver deposition process [54]. To conclude, the deposition of silver was more efficient with a lower deposition solution volume of 4 mL in a glass tank, and by heating the deposition solution with a water bath to a temperature of 35 °C.

### 3.2. Effect of Blocking Treatment on the Silver-Layer Stability

**Figure 4:**
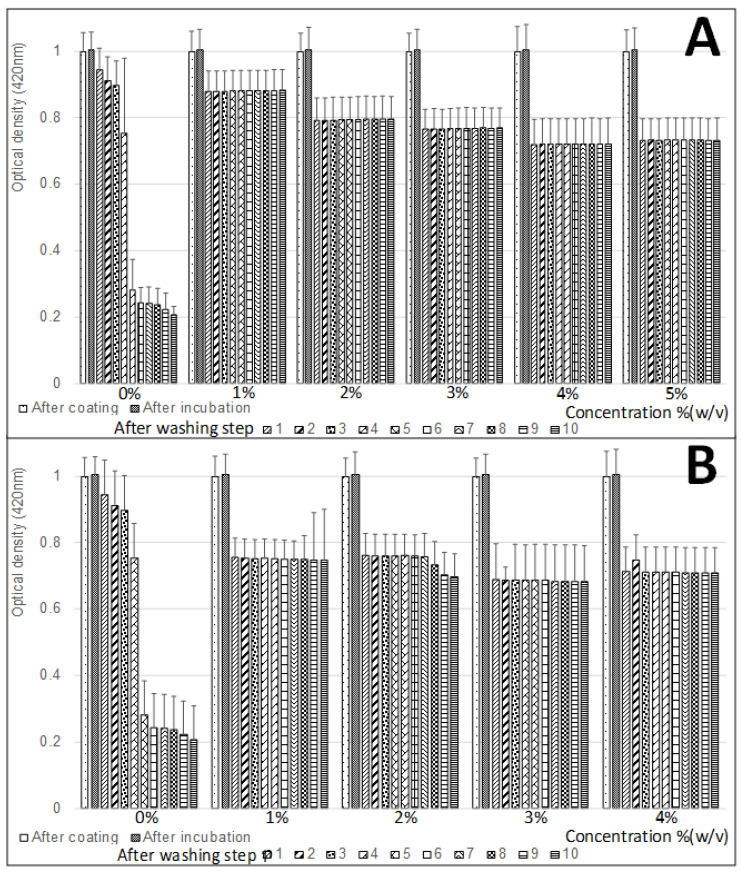
Effect of blocking treatment on the silver-layer stability. The influence of two blocking agents ((**A**) skim milk and (**B**) bovine serum albumin (BSA)) on the silver-layer stability was examined.

A silver layer on a glass surface is susceptible to oxidation [55] as well as to degradation by moisture [56]. Coatings of different materials were previously tested in order to increase the stability of a formed silver layer [57,58,59,60,61,62]. The influence of blocking agent treatment on the stability of the silver layer was also examined. Two blocking agents including skim milk (1, 2, 3, 4 and 5% (*w*/*v*)) and bovine serum albumin (BSA) (1, 2, 3 and 4% (*w*/*v*)) were tested (Figure 4). After the modified glass tubes were treated with different concentrations of the blocking agent solutions, they were rinsed ten times, and the OD_420 nm_ was detected after each washing step as a measure of the silver-layer stability. From the results, generally for both blocking agents in all the tested concentrations, an increase in the silver-layer stability was identified. In the cases of untreated surfaces (Figure 4A,B at 0%), the repeated washing steps caused a 5-times reduction of the silver layer, which was indicated by the reducing patterns of the OD_420 nm_ values from 1 to 0.2. The washing scraped down the already-existing silver layer that was deposited on the glass surface. While, in the cases of the treated surfaces with both blocking agents at all the tested concentrations, the OD_420 nm_ values were consistent after the second washing step, remaining constant between 0.7 to 0.9 in the case of skim milk and 0.7 to 0.75 in the case of BSA. These results indicate that the treatment of a blocking agent protected and increased the silver-layer stability by 4.5-times (0.9/0.2) and 3.75-times (0.75/0.2) in the cases of skim milk and BSA, respectively. This phenomenon can be explained by the absorption of the blocker agent molecules above the deposited silver layer, either on the glass, silver, or surface edges. Moreover, the addition of a blocker agent layer may assist to stabilize the silver layer by creating additional connection forces between the fixed metal and glass surface. The creation of such a blocking agent layer can be observed in the results by the decreasing pattern of the OD_420 nm_ values after the blocking treatment step. In addition, in the comparison between the two blocking agents, the skim milk treatment (OD_420 nm_ = 0.7–0.9) protected more on the silver layer than the BSA treatment (OD_420 nm_ = 0.7–0.75), meaning that, in the case of the BSA treatment, more silver was scraped down from the deposited layer. Interestingly, a decreasing pattern was also identified among the increasing concentrations of skim milk treatment. Higher concentrations of skim milk resulted in lower OD_420 nm_ values (skim milk (*w*/*v*) 1%, 0.9; 2%, 0.8; 3%, 0.75; 4%, 0.7; and 5%, 0.7), indicating less silver-layer stability, which can be explained by its nature to aggregate, therefore it may cause the removal of the silver. To conclude, the treatment of the blocking agent after silver deposition shows a protective effect over the washing steps, which may increase the silver-layer stability.

### 3.3. Effect of the Blocking Step on the Nonspecific DNA Absorption

**Figure 5:**
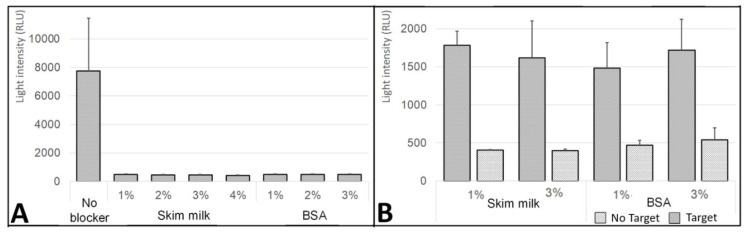
Effect of the blocking step on the DNA nonspecific absorption on the glass surface. The blocking treatment was conducted on the silver-modified glass surfaces, with either bovine serum albumin (BSA) or skim milk. Then, the light signal was compared with the untreated (without blocking agent) glass tubes. After the blocking step, all the tubes were incubated for 1 h with 20 µL 1 µM of the reporter DNA strand linked to horseradish peroxidase (HRP). The light signal was measured after washing, by adding a mixture of 1:1 (*v*/*v*) H_2_O_2_ + luminol solution (HRP substrate). (**A**) Glass tubes without immobilized surface DNA. (**B**) The effect of the blocking step on the annealing processes was examined by conducting the blocking treatment on glass tubes with immobilized 20 µL 1 µM surface DNA strand. Then, they were exposed to the reporter strand (DNA-HRP) with or without 20 µL 1 µM of the target DNA strand. After the annealing process, the glass tubes were washed, and the light signal was measured by adding the H_2_O_2_ + luminol solution (HRP substrate).

The influence of the blocking treatment on the nonspecific DNA absorption was also tested (Figure 5). The main aim of this examination step was to identify the preferred blocking agent to treat the silver-deposited glass surface, which prevents nonspecific DNA binding as well as improves the efficiency of DNA hybridization. It is challenging to theoretically select the preferred blocking agent, therefore, an empirical examination for screening was conducted. Two blocking agents were tested including skim milk (1, 2, 3, and 4% (*w*/*v*)) and BSA (1, 2, and 3% (*w*/*v*)). The silver-deposited glass surface without immobilized surface DNA strand was then incubated with the different blocking solutions. Then, the tubes were exposed to 20 µL 1 µM of the reporter DNA strand for 1 h. After washing and addition of HRP substrate (H_2_O_2_ + luminol) the light signal was detected (Figure 5A). From the results, generally the nonspecific DNA absorption was higher in the case of unblocked (untreated) silver-deposited glass surface as compared to the blocked surface, which can be seen by the higher light signal detected. For all the tested concentrations of the two blocking agents, skim milk and BSA, the blocked (treated) silver-deposited glass tubes (~500 relative light units (RLU)) showed 16-times lower light-signal levels than the unblocked glass tubes (~8000 RLU). The nonspecific DNA absorption that was characterized in the unblocked silver-deposited glass surface may result in increased levels of false-positive response during further measurements. Therefore, the blocking treatment of silver-deposited glass surface is essential in order to allow for a correct biosensor performance in cases of DNA detection. Moreover, the effect of the blocking treatment on the annealing process of mRNAs was examined. The silver-deposited glass surface with immobilized 20 µL 1 µM surface DNA strand was then incubated with the different blocking solutions. Then, the tubes were exposed to 20 µL 1 µM of the reporter DNA strand for 1 h with or without 20 µL 1 µM of the target DNA strand. After washing and addition of HRP substrate (H_2_O_2_ + luminol) the light signal was detected (Figure 5B). The blocking solutions at the tested concentrations not only did not cause any disturbance to the annealing process, but it also increased the efficiency of the annealing process by 4.38-times (with target DNA: 1500–1750 RLU vs. without target DNA: 400–500 RLU). To conclude, the patterns of the uniform results indicate that the blocking treatment at all the tested concentrations resulted in a similar blocking-layer formation that reduced the nonspecific DNA absorption to the silver-deposited glass surface and did not cause any disturbance to the annealing process of mRNAs.

### 3.4. Effect of the Deposition-Reaction Duration on the Silver-Layer Formation and the Metal-Enhanced Fluorescence (MEF) Amplification

**Figure 6:**
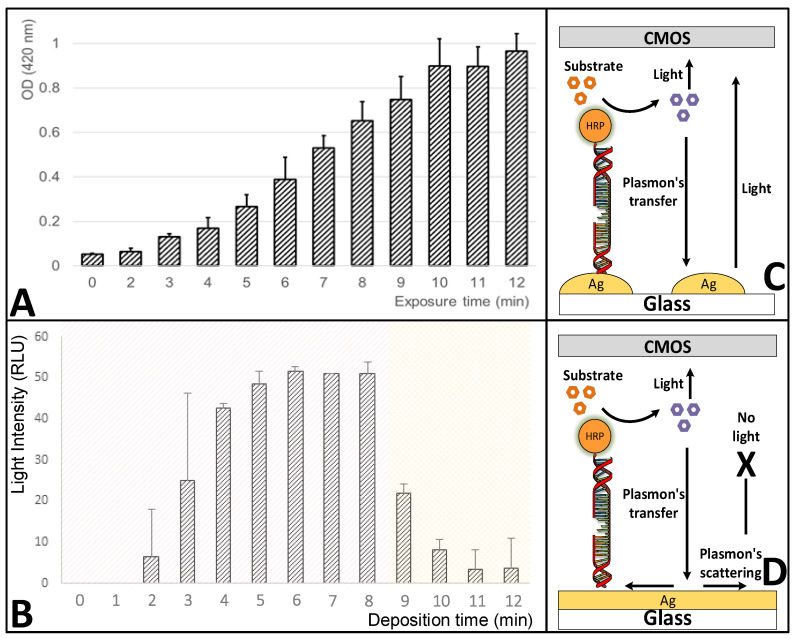
Effect of the deposition reaction duration on the silver-layer formation and the metal-enhanced fluorescence (MEF) amplification. Monitoring the influence of the silver-deposition reaction duration on both (**A**) optical density at 420 nm (OD_420 nm_), and (**B**) relative light units (RLU). (**C**) With a distance lower than 10 nm between the silver-deposited glass surface and the light photons that were generated from the horseradish peroxidase (HRP) enzyme reaction with its substrate, a plasmons transfer occurred that increased the light signal, which was later detected by the complementary metal-oxide-semiconductor (CMOS) sensor. (**D**) After excessive silver deposition the silver dots formed a silver layer, and the plasmons scattered and reduced the light signal.

**Figure 7:**
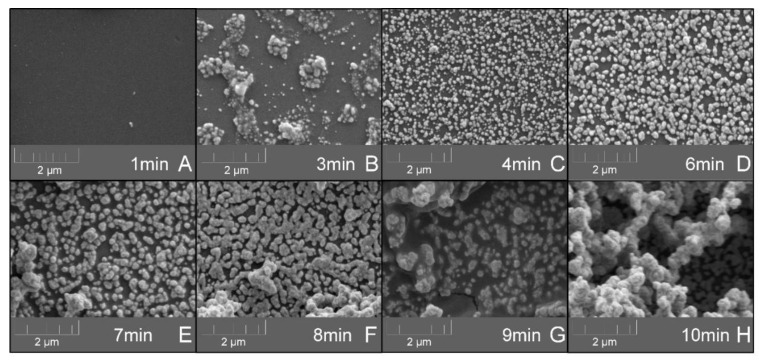
Scanning electron microscopy (SEM) characterization of the effect of the deposition time on the formation of silver nano-islands. The surface morphology was characterized by an SEM model MIRA3 from TESCAN (Brno-Kohoutovice, Czech Republic), at a 5 kV accelerating voltage. Before imaging, a thin layer of palladium gold was deposited onto the samples in order to render them electrically conductive and to avoid potential surface charging by the electron beam. The influence of the silver-deposition reaction duration of (**A**) 1 min; (**B**) 3 min; (**C**) 4 min; (**D**) 6 min; (**E**) 7 min; (**F**) 8 min; (**G**) 9 min; and (**H**) 10 min, was investigated. A clear correlation is visible between the deposition time and the size and amount of the silver nano-islands. Longer deposition time resulted in significantly-larger silver nano-islands (8 > 7 > 5 > 4 min).

The developed biosensor platform is based on the MEF phenomenon, which was amplified by the deposition of a silver layer onto a glass surface [63]. The silver-deposition procedure is a kinetic reaction and the formation of a silver layer is highly dependent on the deposition reaction duration [64,65]. The MEF phenomenon is utilized in various biosensor platforms in order to increase their sensitivity and performance [66] and also in cases of RNA detection [67]. The design of micro- and nano-metal surface details are used in order to enhance or quench fluorescence signals [65]. MEF is described as the interaction of fluorophores with a metallic surface within a 5–90 nm distance and has enhancing effects on optical properties such as quantum yield, photostability, and lifetime of fluorophores [68]. The effect of the silver-deposition reaction duration on the silver-layer formation was examined, and most importantly its effect on the MEF amplification (Figure 6). Except for the reaction duration, the rest of the procedure parameters remained constant, as well as the concentration of the reporter DNA linked to the HRP enzyme (100 nM). Over a silver-deposition reaction duration ranging between 0 and 12 min, both the OD_420 nm_ and the RLU signals were monitored. Generally, with a distance lower than 10 nm [69] between the silver-deposited glass surface and the light photons that are generated from the HRP enzyme reaction with its substrate, a plasmons transfer occurs that increases the light signal, which is later detected by the CMOS sensor. After excessive silver deposition, when the silver dots form a silver layer, the plasmons scatter and reduce the light signal [70]. From the results, it is clear that in a longer deposition reaction duration, more silver was deposited on the glass surface. The OD_420 nm_ values increased by 20-times over the increasing reaction duration from 0.05 (t = 0) and up to 1 (t = 12 min) (Figure 6A). A previous study also reported that by increasing the deposition reaction duration a more uniform silver layer was formed [71]. While, as expected, the RLU values showed an increasing pattern that then shifted into a decreasing pattern. The RLU values firstly increased from 0 (t = 0) to 50 RLU (t = 5–8 min), and then reduced to 5 RLU (t = 12 min) (Figure 6B). In a silver-deposition reaction duration of up to 8 min, the plasmons from the active optical molecules transferred to the silver nano-dots on the glass tube surface and stimulated the light signal response, resulting in the increasing RLU values (Figure 6C), whereas, afterward in the silver-deposition reaction duration between 9 and 12 min, the glass surface was covered with a uniform (monolith) silver layer, which caused the scattering of plasmons, and instead of producing light induction they scattered on the silver surface, resulting in the decreasing RLU values (Figure 6D). The identification of this shift in the MEF phenomenon is highly important for the enhancement of the light signal that is later detected by the CMOS sensor. It is needed to identify the exact preferred silver-deposition reaction duration, in order to allow for the increase in the number of silver nano-dots, while still prevent the creation of a monolith silver layer because further increase of the silver-deposition reaction duration will result in the reduction of the number of separate (isolated) silver nano-dots, and therefore, it will reduce the MEF light signal enhancement effect. Over the tested silver-deposition reaction duration, a steady-state period was identified within a deposition time of 5 to 8 min that produced a relatively-sTable 50 RLU light signal (Figure 6B). In this period, the plasmons scattering effect was compensated by a possible increase in the concentration of the immobilized DNA. In addition, SEM characterization was conducted on the effect of the deposition time on the formation of silver nano-islands (Figure 7). The results enforce the prior findings, where a clear correlation was visible between the deposition time and the size and amount of the silver nano-islands. Longer deposition time resulted in significantly-larger silver nano-islands (8 > 7 > 5 > 4 min). A monolayer was formed at a deposition time of 9 min, which also confirms the reason behind the decrease in the light signal response. From a deposition time of 7 min onwards, 3D formations were already visible, which is a possible explanation for the steady-state period in the light signal response with a 50 RLU light signal (Figure 6B). This finding also enforces the assumption that in this deposition time-period the plasmons scattering effect was compensated by a possible increase in the concentration of the immobilized DNA, because the 3D constructions may have resulted in increased molecule immobilization, while still resulting in a monolayer surface that reduced the overall light response by the scattering process. To conclude, a preferred silver-deposition reaction duration of 5 to 8 min was identified, which increased the deposition of silver on the glass surface by up to 13-times, and also resulted in the amplification of the MEF phenomenon and the produced light signal to the maximum signal of 50 RLU that was later detected by the CMOS sensor.

### 3.5. The Sensitivity of the CMOS-Based Biosensor System to Colletotrichum gloeosporioides Fungi

**Figure 8:**
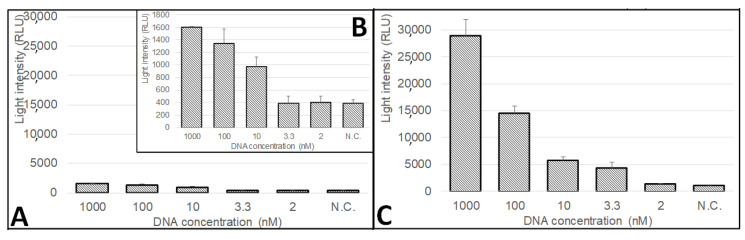
The sensitivity of the CMOS-based biosensor system to *Colletotrichum gloeosporioides* fungi. The response of the biosensor system to different concentrations of the quiescent marker of *Colletotrichum gloeosporioides*, a specific sequence of enoyl-CoA-hydratase/isomerase (Cgl_00014454), before and after integrating the conclusions from the optimization steps of the biosensor procedures. (**A** + **B**) initial protocol and (**C**) improved protocol. Negative control (N.C.) was used. For both protocols, the modified glass surfaces were immobilized with 20 µL 1 µM surface DNA strand, exposed to the target DNA strand in different concentrations, and later exposed to 20 µL 1 µM quiescent-stage reporter DNA strand.

The sensitivity of the developed CMOS-based biosensor system was lastly examined for the detection of the quiescent DNA marker of *Colletotrichum gloeosporioides*, a specific sequence of enoyl-CoA-hydratase/isomerase (Cgl_00014454) (Figure 8). The pathogenic fungus usually penetrates the fruit before harvest. Then, it enters into a quiescent and microscopic phase, and only when the fruit ripens does the fungi switch to its necrotrophic phase and cause decay [13]. The enoyl-CoA-hydratase/isomerase gene is highly upregulated during the fungal quiescent state, while other metabolic and transcriptomic activities are significantly reduced [14]. DNA strands have been widely used in order to detect fungi, bacteria, and other genetically-modified organisms [72]. In this study, a complementary CMOS-based biosensor was developed, which integrated specific DNA strands that allowed the detection of enoyl-CoA-hydratase/isomerase. The conclusions that were discussed in the previous sections were integrated in order to form an improved silver-deposition procedure protocol. Then, a comparison of the detection sensitivity was conducted between two setups that were based on the initial or the improved protocols. The initial procedure included a 24-mL silver reaction solution heated at 35 °C on the heater plate for 9 min. While in the improved protocol, the 4-mL silver reaction solution was heated at 35 °C in a water bath for 5 min. Then, the glass tubes were immobilized with 20 µL 1 µM surface DNA strands and then treated with a blocking agent solution of 1% (*w*/*v*) skim milk. For both the initial and improved protocols, the modified glass surfaces were then exposed to the target DNA strand in different concentrations, and later exposed to 20 µL 1 µM quiescent-stage reporter DNA strand. From the results, the biosensor demonstrated the ability to distinguish between the different concentrations of the target DNA for up to a 10 nM threshold (Figure 8A,B). The implementation of the improved procedure increased the biosensor sensitivity to 3.3 nM, by using the preferred number of silver nano-dots deposited on the glass surface (Figure 8C). Moreover, the light signal was amplified in the improved protocol (1000 nM, 30,000 RLU; 100 nM, 15,000 RLU; 10 nM, 5500 RLU; 3.3 nM, 4500 RLU; and 2 nM, 1500 RLU) as compared to the initial protocol (1000 nM, 1600 RLU; 100 nM, 1350 RLU; 10 nM, 1000 RLU; 3.3 nM, 400 RLU; and 2 nM, 400 RLU) for all the tested concentrations. The light signal was higher by 18.75-, 11.11-, 5.5-, 11.25-, and 3.75-times in the improved protocol for all the tested concentrations of the target DNA strand of 1000, 100, 10, 3.3, and 2 nM, respectively. The developed CMOS-based biosensor system demonstrated comparable detection sensitivity with other optical-based [73,74], electrochemical-based [75,76], and mass-balance-based [77,78] biosensor platforms for pathogen detection in plants. The PCR technology demonstrates a more sensitive DNA detection; however, it is not suitable for real-time monitoring on agricultural sites. To conclude, MEF can be amplified by a customized silver-deposition procedure that results in increased detection sensitivity.

## 4. Conclusions

In this study, a CMOS-based biosensor was developed, which integrates specific DNA strands that allow the detection of enoyl-CoA-hydratase/isomerase, which is the quiescent marker of *Colletotrichum gloeosporioides* fungi. The developed biosensor mechanism is based on the MEF phenomenon, which is amplified by depositing silver onto a glass surface. Several parameters that affect the silver-deposition procedure were examined, including silver solution temperature and volume, heating mode, and the tank material. From the results, the deposition of silver was more efficient with a lower deposition solution, a volume of 4 mL in a glass tank, and by heating the deposition solution with a water bath to a temperature of 35 °C. Moreover, the effect of blocking treatment (skim milk or BSA) on the silver-layer stability and nonspecific DNA absorption was tested. It was found that the treatment of a blocking agent after silver deposition shows a protective effect over washing steps, which may increase the silver-layer stability. The patterns of the uniform results indicate that the blocking treatment at all the tested concentrations resulted in a similar blocking-layer formation that reduced the nonspecific DNA absorption to the silver-deposited glass surface. The effect of the deposition reaction duration on the silver-layer formation and the MEF amplification was also investigated. A preferred silver-deposition reaction duration was identified of 5 to 8 min, which increased the deposition of silver on the glass surface and resulted in the amplification of the MEF phenomenon and the produced light signal that was later detected by the CMOS sensor. It was found that MEF can be amplified by a customized silver-deposition procedure that results in increased detection sensitivity. The implementation of the improved conditions increased the biosensor sensitivity to 3.3 nM (4500 RLU) with a higher detected light signal as compared to the initial protocol (400 RLU). Moreover, the light signal was amplified by 18.75-, 11.11-, 5.5-, 11.25-, and 3.75-times in the improved protocol for all the tested concentrations of the target DNA strand of 1000, 100, 10, 3.3, and 2 nM, respectively. Several previous studies described the development of CMOS-based biosensor systems for the detection of nucleic acids [79,80]. However, they mostly address the detection of pathogens in healthcare [24,81], and not in agriculture or food. The developed biosensor system demonstrated reproducible results within the same setup as well as between different setups. The developed biosensor may allow the detection of the pathogenic fungus in postharvest produce and determine its pathogenicity state.
